# Supplementary material for: A New Fluorescence Detection Method for Tryptophan- and Tyrosine-Derived Allelopathic Compounds in Barley and Lupin
Source: Plants (Basel). 2023 May 9;12(10):1930. doi: 10.3390/plants12101930 (PMC10222917; doi:10.3390/plants12101930)
Supplement: Supplementary file 1 [file plants-12-01930-s001.zip › plants-2334504-supplementary.pdf]

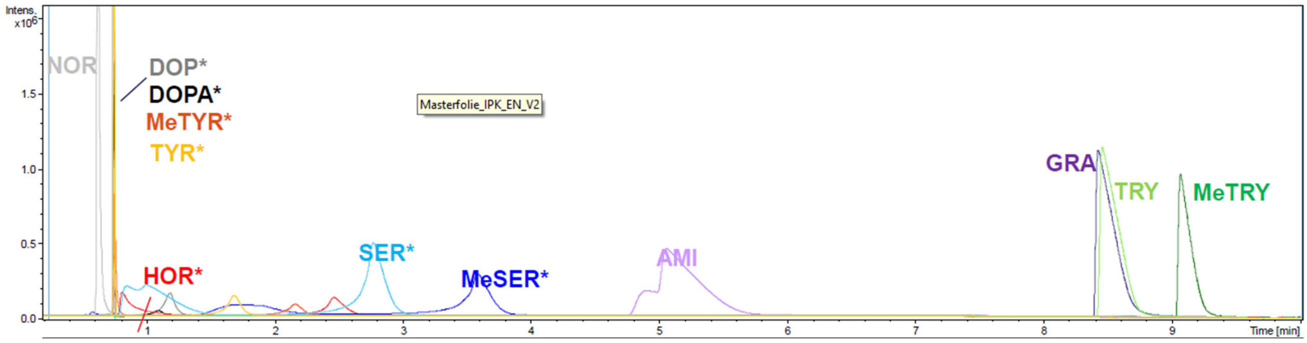

Figure S1: Representative chromatogram of the initial tests performed with the HSS T3 (100 A, 1.8 uM, 2.1 x 100 mm) stationary phase with a mix of the following standards: noradrenaline (NOR), dopamine hydrochloride (DOP), L-3-Hydroxytyrosine (DOPA), tyramine (TYR), -methyl-tyramine (MeTYR), hordenine (HOR), serotonin (SER), N-Methyl-serotonin (MeSER), 3-aminomethylindole (AMI), gramine (GRA), tryptophan (TRP) and *N*-methyltryptamine (MeTRY).

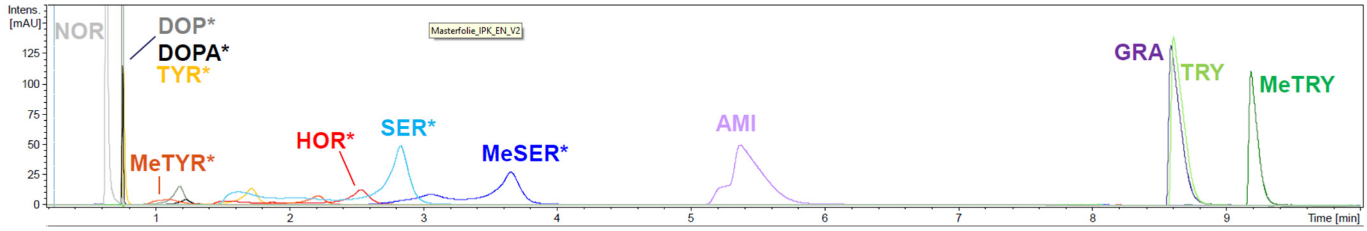

Figure S2: Evolution of the appearance of the chromatogram of the tests performed with the HSS T3 (100 A, 1.8 uM, 2.1 x 100 mm) stationary phase with a mix standards (noradrenaline (NOR), dopamine hydrochloride (DOP), L-3-Hydroxytyrosine (DOPA), tyramine (TYR), -methyl-tyramine (MeTYR), hordenine (HOR), serotonin (SER), N-Methyl-serotonin (MeSER), 3-aminomethylindole (AMI), gramine (GRA), tryptophan (TRP) and *N*-methyltryptamine (MeTRY)) after the increase of the formic acid from 0.1 to 0.5%.

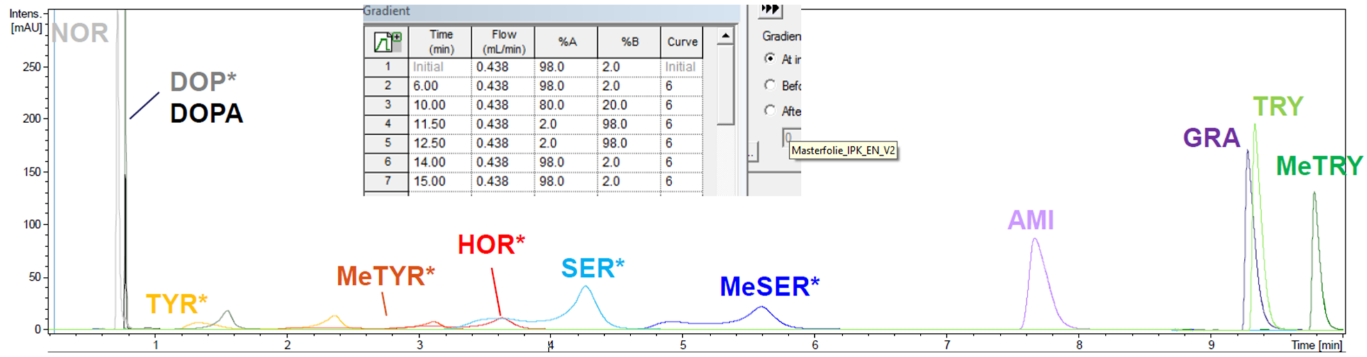

Figure S3: Evolution of the appearance of the chromatogram of the tests performed with the HSS T3 (100 A, 1.8 uM, 2.1 x 100 mm) stationary phase with a mix standards (noradrenaline (NOR), dopamine hydrochloride (DOP), L-3-Hydroxytyrosine (DOPA), tyramine (TYR), -methyl-tyramine (MeTYR), hordenine (HOR), serotonin (SER), N-Methyl-serotonin (MeSER), 3-aminomethylindole (AMI), gramine (GRA), tryptophan (TRP) and *N*-methyltryptamine (MeTRY)) after modification of the eluents (10 mM ammonium acetate (pH 5.0) (A) and acetonitrile (B)).

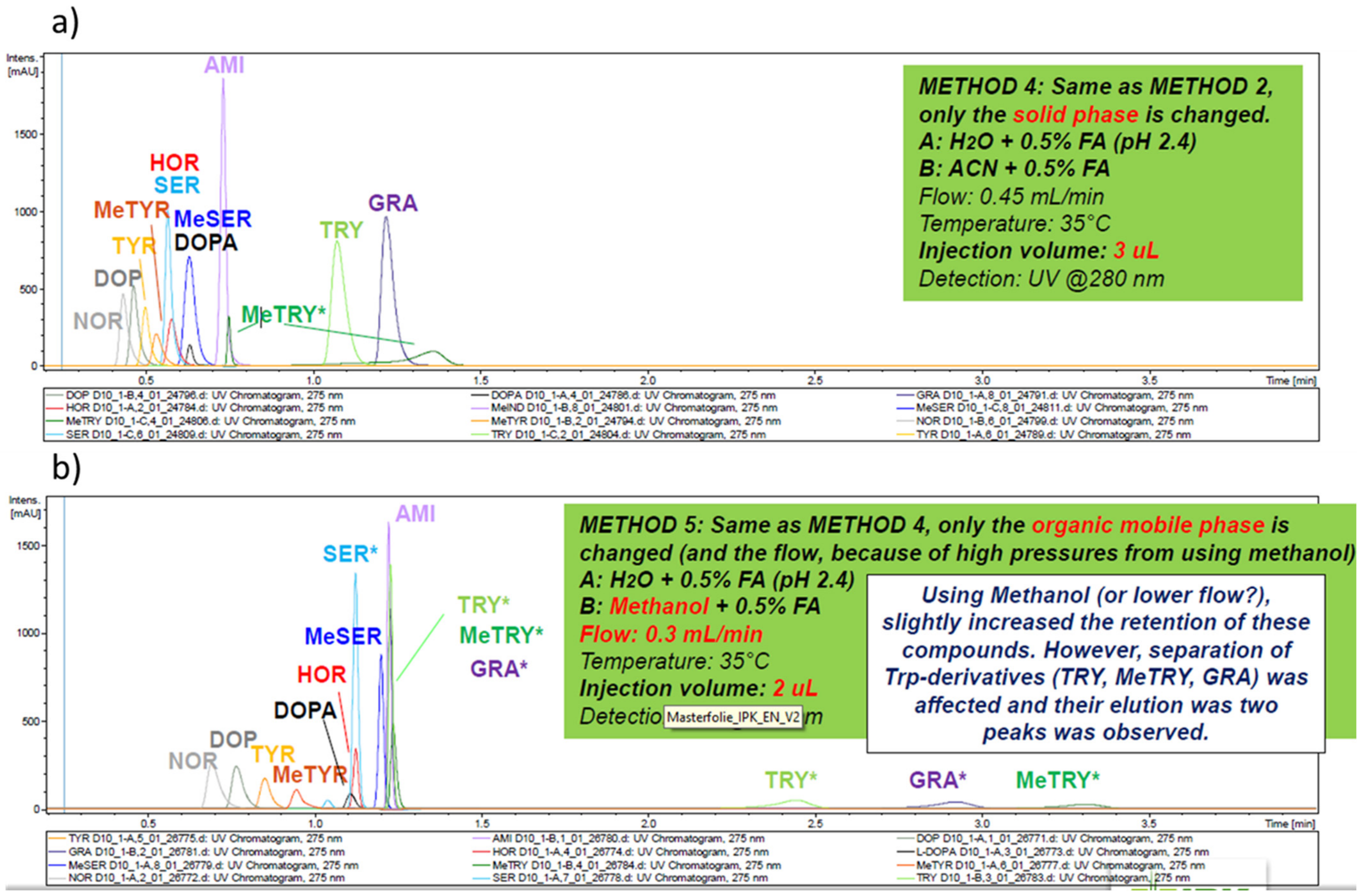

Figure S4: Representative chromatograms obtained with a CSH phenyl-hexyl (130 A, 1.7 uM, 2.1 x 100 mm) in combination with different mobile phases. a) chromatogram obtained with the mobile phase being constituted by water with the addition of 0.5% of formic acid (pH 2.4) (A) and acetonitrile with the addition of 0.5% of formic acid (B); b) chromatogram obtained with the mobile phase being constituted by water with the addition of 0.5% of formic acid (pH 2.4) (A) and methanol with the addition of 0.5% of formic acid (B). Standards: noradrenaline (NOR), dopamine hydrochloride (DOP), L-3-Hydroxytyrosine (DOPA), tyramine (TYR), -methyl-tyramine (MeTYR), hordenine (HOR), serotonin (SER), N-Methyl-serotonin (MeSER), 3-aminomethylindole (AMI), gramine (GRA), tryptophan (TRP) and *N*-methyltryptamine (MeTRY).

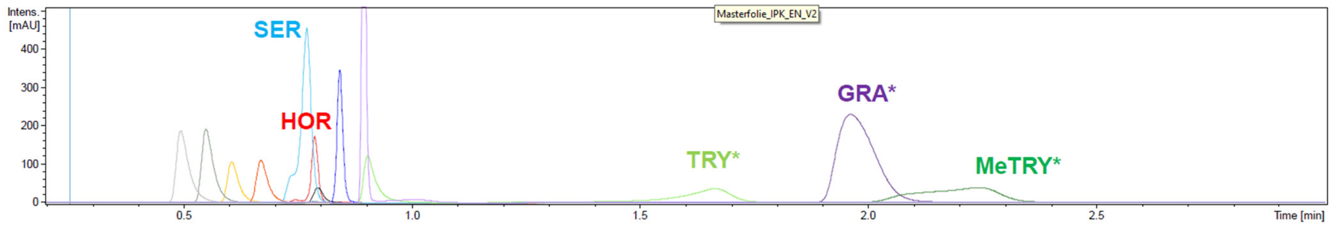

Figure S5: Representative chromatograms obtained with a CSH phenyl-hexyl (130 A, 1.7 uM, 2.1 x 100 mm) after the increase of the proportion of aqueous mobile phase in the isocratic flow and lowering the flow rate. The run was performed with the following standard mix: noradrenaline (NOR), dopamine hydrochloride (DOP), L-3-Hydroxytyrosine (DOPA), tyramine (TYR), -methyl-tyramine (MeTYR), hordenine (HOR), serotonin (SER), N-Methyl-serotonin (MeSER), 3-aminomethylindole (AMI), gramine (GRA), tryptophan (TRP) and *N*-methyltryptamine (MeTRY).

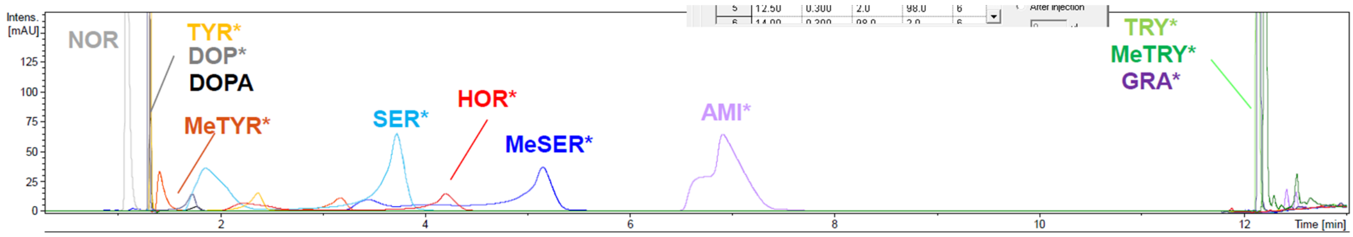

Figure S6: Representative chromatograms obtained with an Acquity UPLC BEH phenyl 130 A, 1.7 uM, 2.1 x 100 mm running a mix of the following standards: noradrenaline (NOR), dopamine hydrochloride (DOP), L-3-Hydroxytyrosine (DOPA), tyramine (TYR), -methyl-tyramine (MeTYR), hordenine (HOR), serotonin (SER), N-Methyl-serotonin (MeSER), 3-aminomethylindole (AMI), gramine (GRA), tryptophan (TRP) and *N*-methyltryptamine (MeTRY), which shows distorted peaks and co-elution.

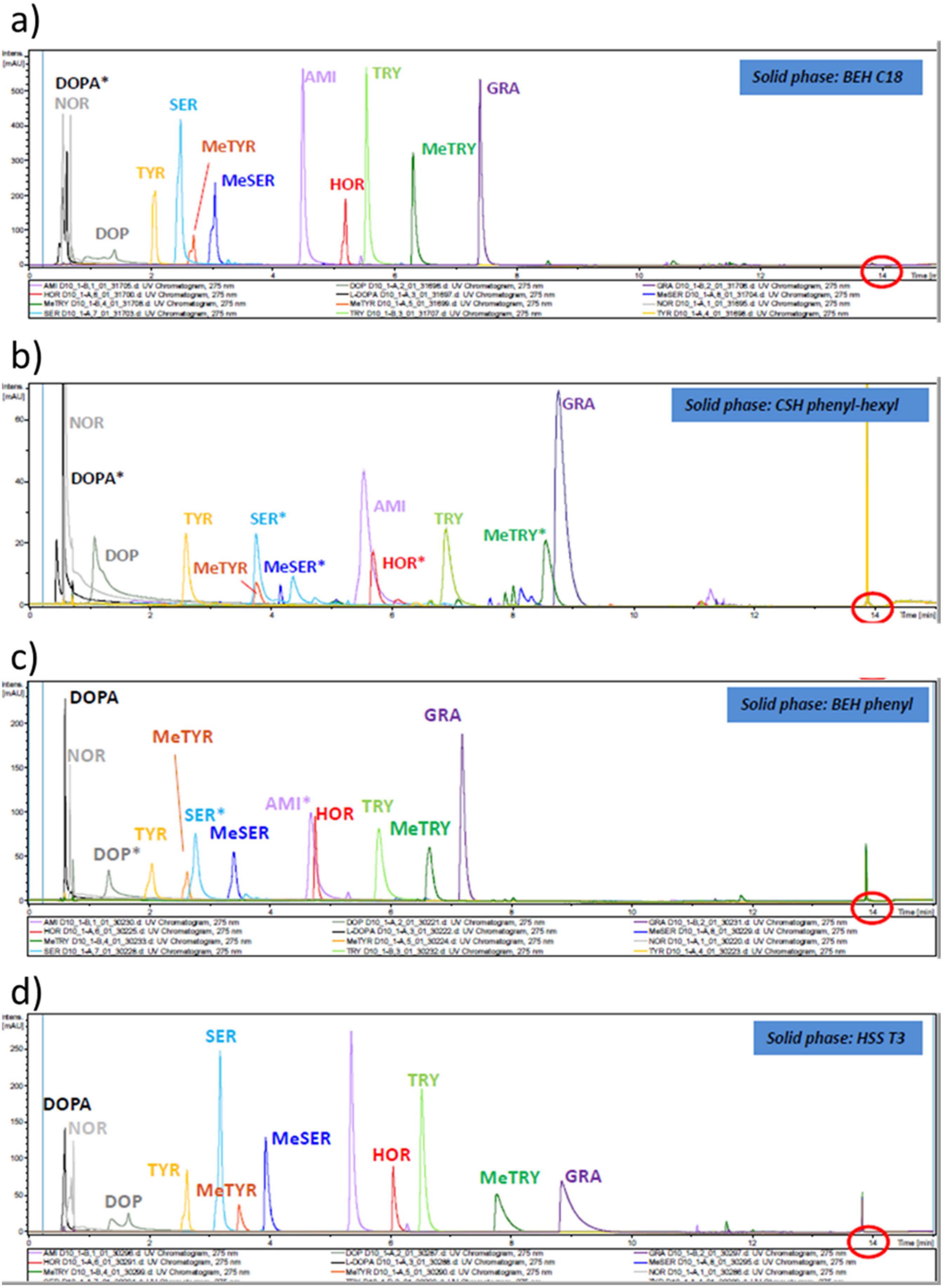
a)
Solid phase: BEH C18
DOPA*
NOR
SER
MeTYR
TYR
MeSER
DOP
AMI
TRY
HOR
MeTRY
GRA
b)
Solid phase: CSH phenyl-hexyl
NOR
DOPA*
DOP
TYR
SER*
MeTYR
MeSER*
AMI
HOR*
TRY
MeTRY*
GRA
c)
Solid phase: BEH phenyl
DOPA
NOR
MeTYR
TYR
SER*
MeSER
DOP*
AMI*
HOR
TRY
MeTRY
GRA
d)
Solid phase: HSS T3
DOPA
NOR
DOP
TYR
MeTYR
SER
MeSER
HOR
TRY
MeTRY
GRA

Figure S7: Comparison between the chromatographic results obtained from the analysis of a mix of standards (noradrenaline (NOR), dopamine hydrochloride (DOP), L-3-Hydroxytyrosine (DOPA), tyramine (TYR), -methyl-tyramine (MeTYR), hordenine (HOR), serotonin (SER), N-Methyl-serotonin (MeSER), 3-aminomethylindole (AMI), gramine (GRA), tryptophan (TRP) and *N*-methyltryptamine (MeTRY)) with various stationary phases. Representative chromatograms of runs performed with a) BEH C18 stationary phase; b) CSH phenyl-hexyl (130 A, 1.7 uM, 2.1 x 100 mm) stationary phase; c) Acquity UPLC BEH phenyl (130 A, 1.7 uM, 2.1 x 100 mm) stationary phase; d) HSS T3 (100 A, 1.8 uM, 2.1 x 100 mm) stationary phase.

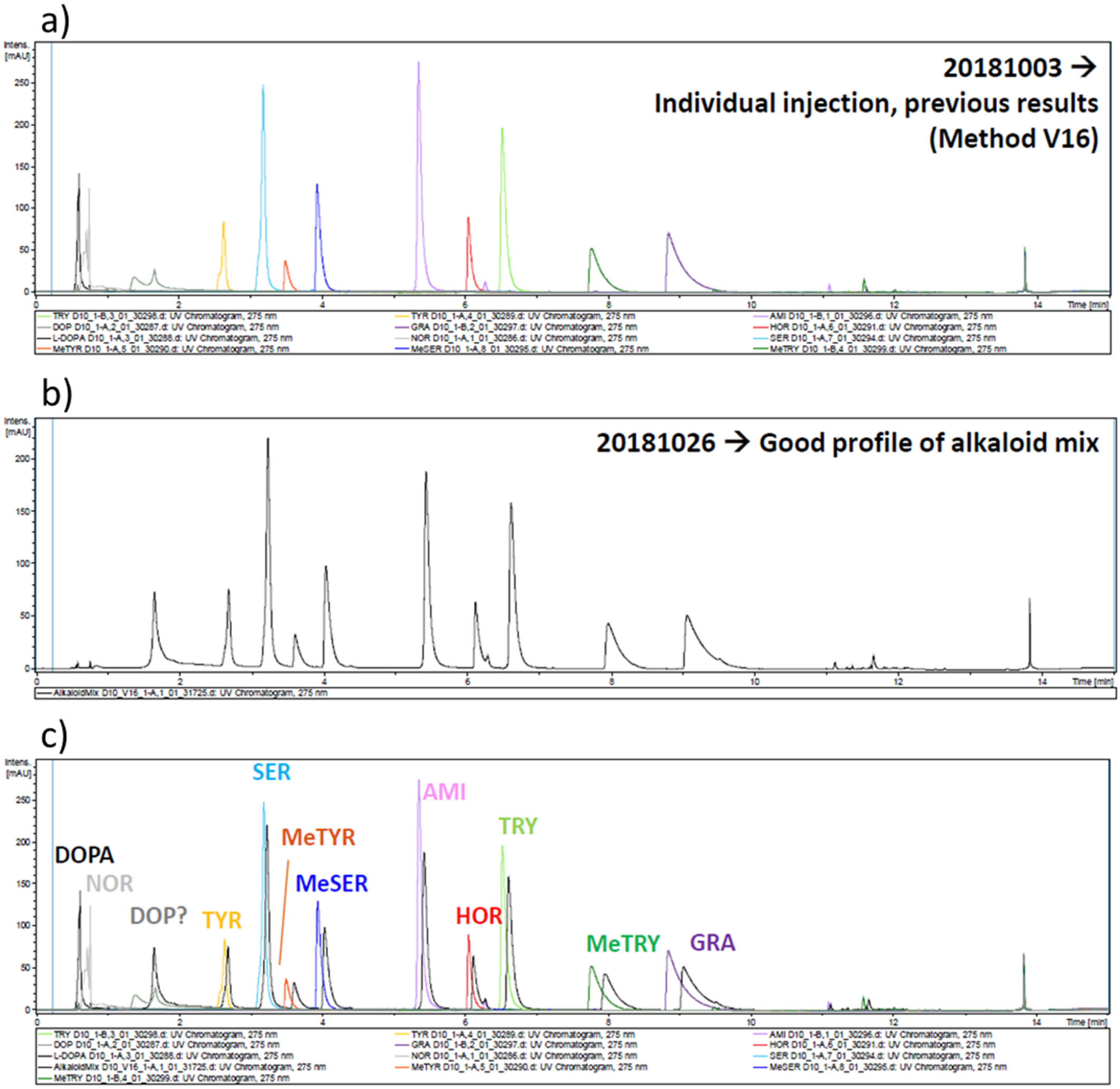

Figure S8: Comparison of the chromatograms obtained from runs performed with HSS T3 (100 A, 1.8 uM, 2.1 x 100 mm) stationary phase upon modification of the pH to 10. Standard mix: noradrenaline (NOR), dopamine hydrochloride (DOP), L-3-Hydroxytyrosine (DOPA), tyramine (TYR), -methyl-tyramine (MeTYR), hordenine (HOR), serotonin (SER), N-Methyl-serotonin (MeSER), 3-aminomethylindole (AMI), gramine (GRA), tryptophan (TRP) and *N*-methyltryptamine (MeTRY). A) Chromatograms resulted from singles standard injections overlayed; b) mix of standards and c) comparison of the chromatograms obtained in a) and b).

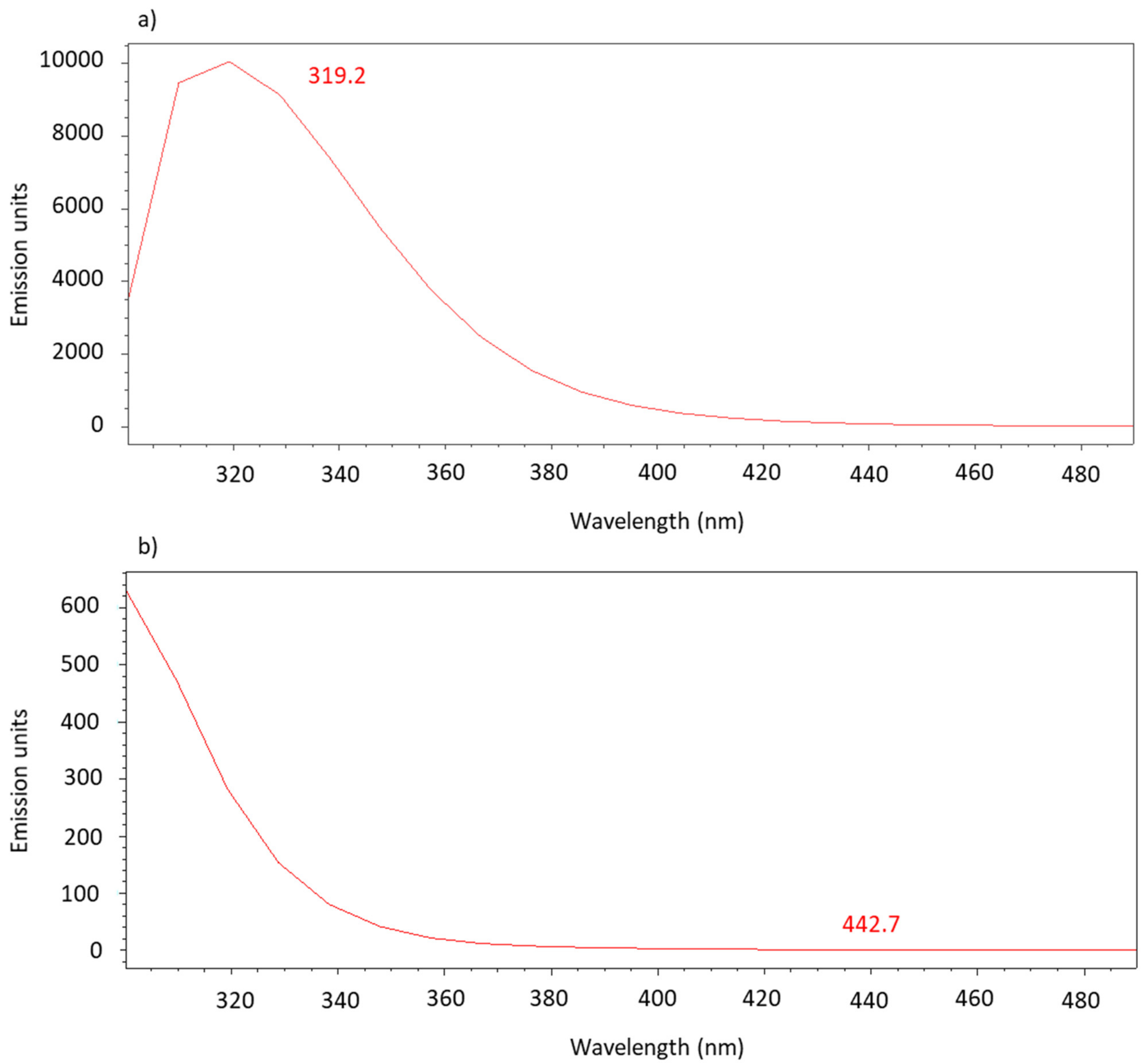

Figure S9: Emission Spectra in response to an excitation wavelength of 280 nm. a) gramine, b) hordenine.

Table S1: Incurred traces left by an unknown contaminant present in the formic acid (n=5).

Injection	Contaminant trace (peak area)
1	242397
2	160556
3	274554
4	264426
5	230223
Mean	234431
Standard deviation	44855
Relative standard deviation	19.2

Table S2: Retention time intervals for Noradrenaline (NOR), dopamine hydrochloride (DOP), tyramine (TYR), hordenine (HOR), 3-aminomethylindole (AMI), tryptophan (TRP), gramine (GRA) and *N*-methyltryptamine (MeTRY).

Compound	Retention time (min)
NOR	0.88 ± 0.04
DOP	2.15 ± 0.18
TYR	3.14 ± 0.19
HOR	3.78 ± 0.22
AMI	5.08 ± 0.30
TRP	5.79 ± 0.28
GRA	6.40 ± 0.70
MeTRY	6.99 ± 0.18

Table S3: Equations for the regression curves of the calibration injections on the UPLC-MS (1) for gramine, hordenine, AMI, tryptophan and tyramine (n=5).

Compound	Equation	SE y-intercept	Coefficient of determination R^2
Gramine (EIC 130.0669)	y= 423319.21x + 606230.75	35275.07	0.9814
Gramine (EIC 175.1239)	y= 62462.00x + 3344.34	3344.34	0.9946
Hordenine	y= 708872.01x + 2619648.07	52974.15	0.9599
AMI	y= 66169.95x + 61459.25	18663.91	0.9937
Tryptophan	y= 163060.74x + 99867.84	16367.02	0.9934
Tyramine	y= 11259.73x + 37944.09	1644.92	0.9857

Table S4: Equations for the regression curves of the calibration injections on the UPLC-FLD (2) for gramine, hordenine, AMI, tryptophan and tyramine (n=5).

Compound	Equation	SE y-intercept	Coefficient of determination R^2
Gramine	y= 8,576,206.94x - 3,712,413.42	21786079.93	0.9994
Hordenine	y= 315,472.41x + 436,311.58	700223.5936	0.9846
AMI	y= 3,249,301.31x + 8,915,277.27	4386915.462	0.9874
Tryptophan	y= 1,111,190.55x + 2,547,912.91	5805055.705	0.9649
Tyramine	y= 297,748.60x + 28,905,245.64	685686.0758	0.9766

Table S5: Limit of detection (LOD) and limit of quantification (LOQ) for gramine, hordenine, AMI, tryptophan and tyramine on UPLC-FLD (2).

	Fluorescence detection (2)	
Compound	LOD (pmol/µL)	LOQ (pmol/µL)
Gramine	8.38	25.40
Hordenine	7.32	22.20
AMI	4.46	13.50
Tryptophan	17.24	52.24
Tyramine	7.60	23.03

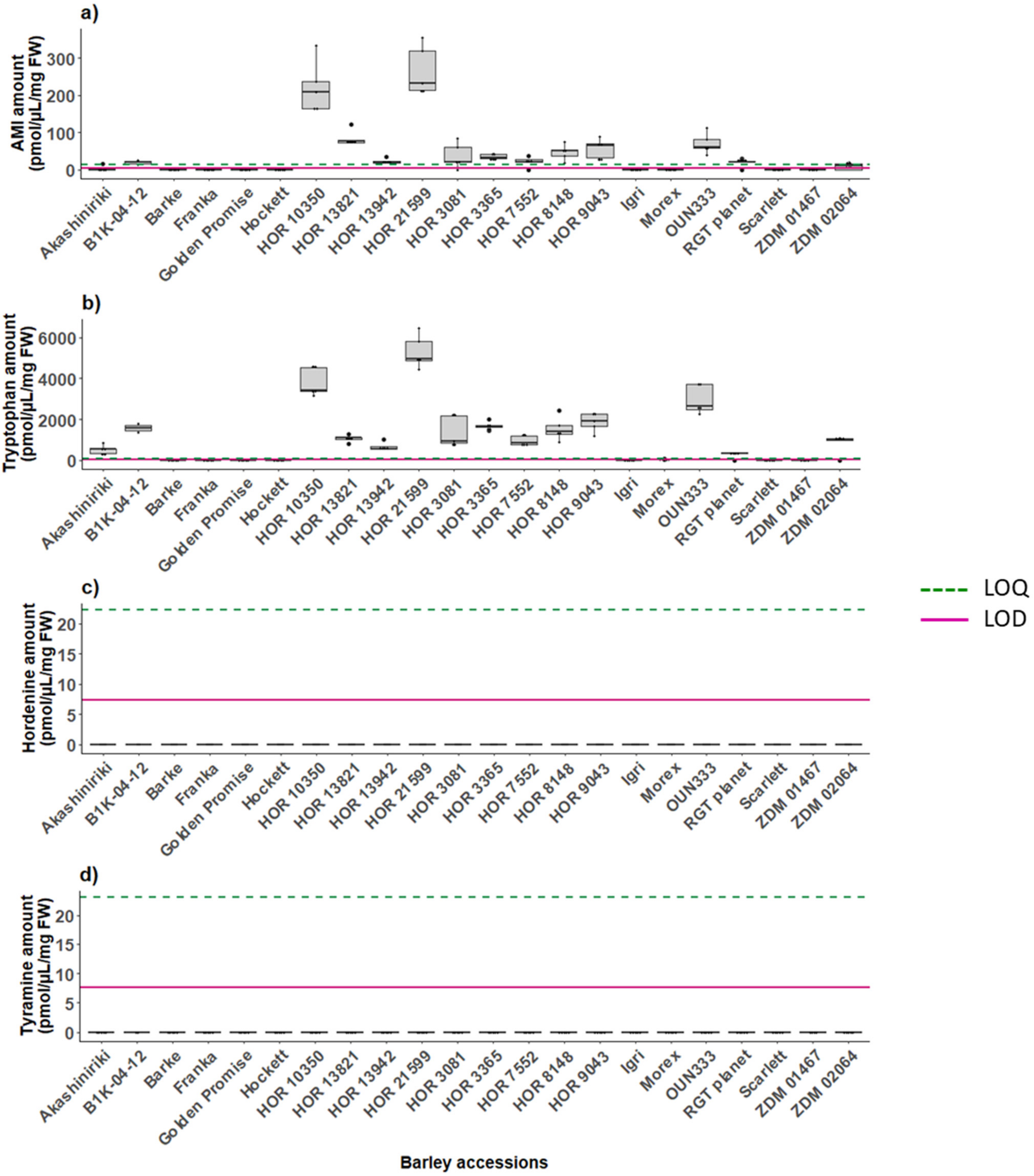

Figure S10: Different analytes production in the leaves 8 DAG among 22 accessions of barley (n=6), a) AMI, b) tryptophan, c) hordenine, d) tyramine. Graphs report the LOD and LOQ values reported in Table S5.

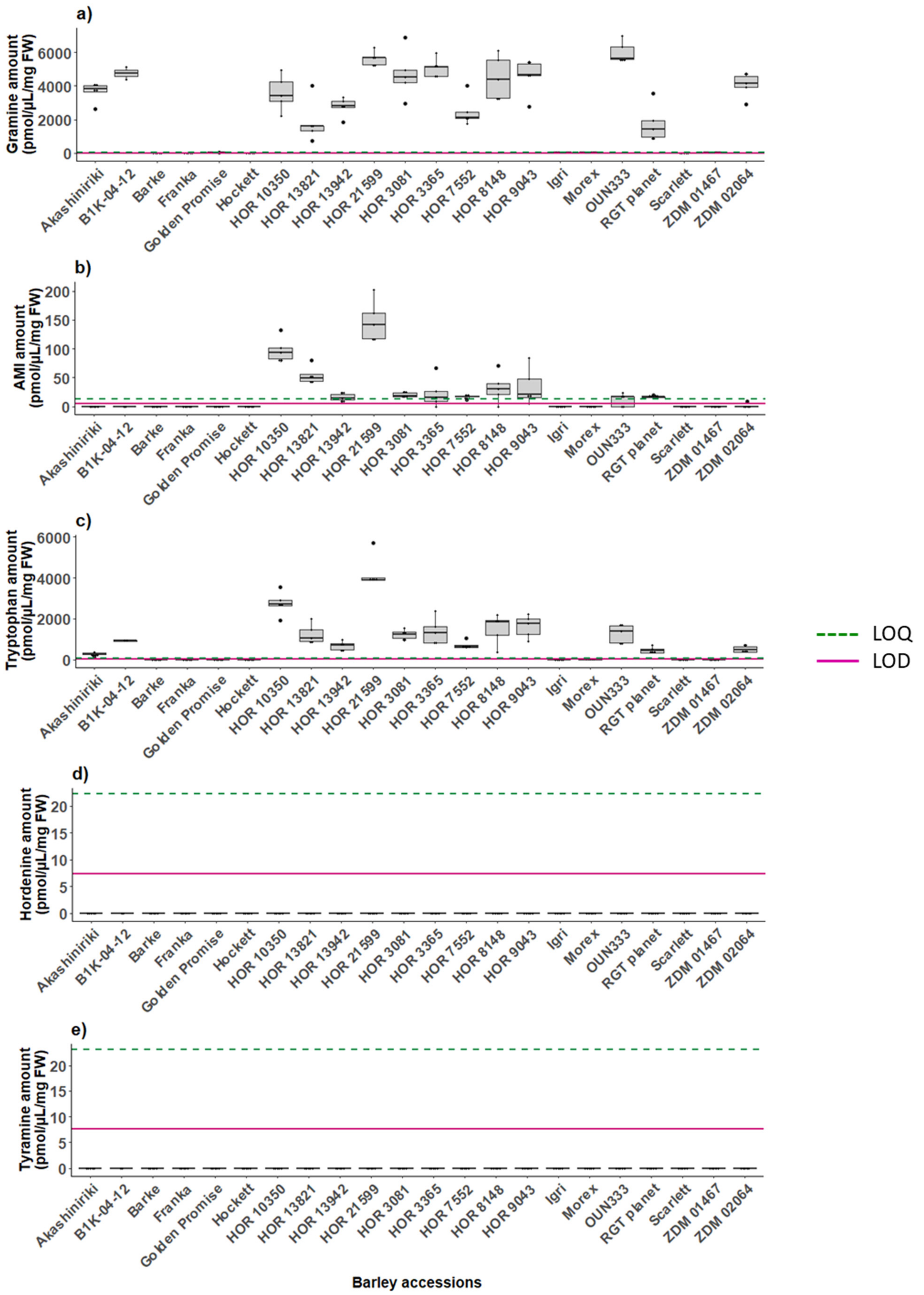

Figure S11: Different analytes production in the leaves 11 DAG among 22 accessions of barley (n=6), a) gramine, b) AMI, c) tryptophan, d) hordenine, e) tyramine. Graphs report the LOD and LOQ values reported in Table S5.

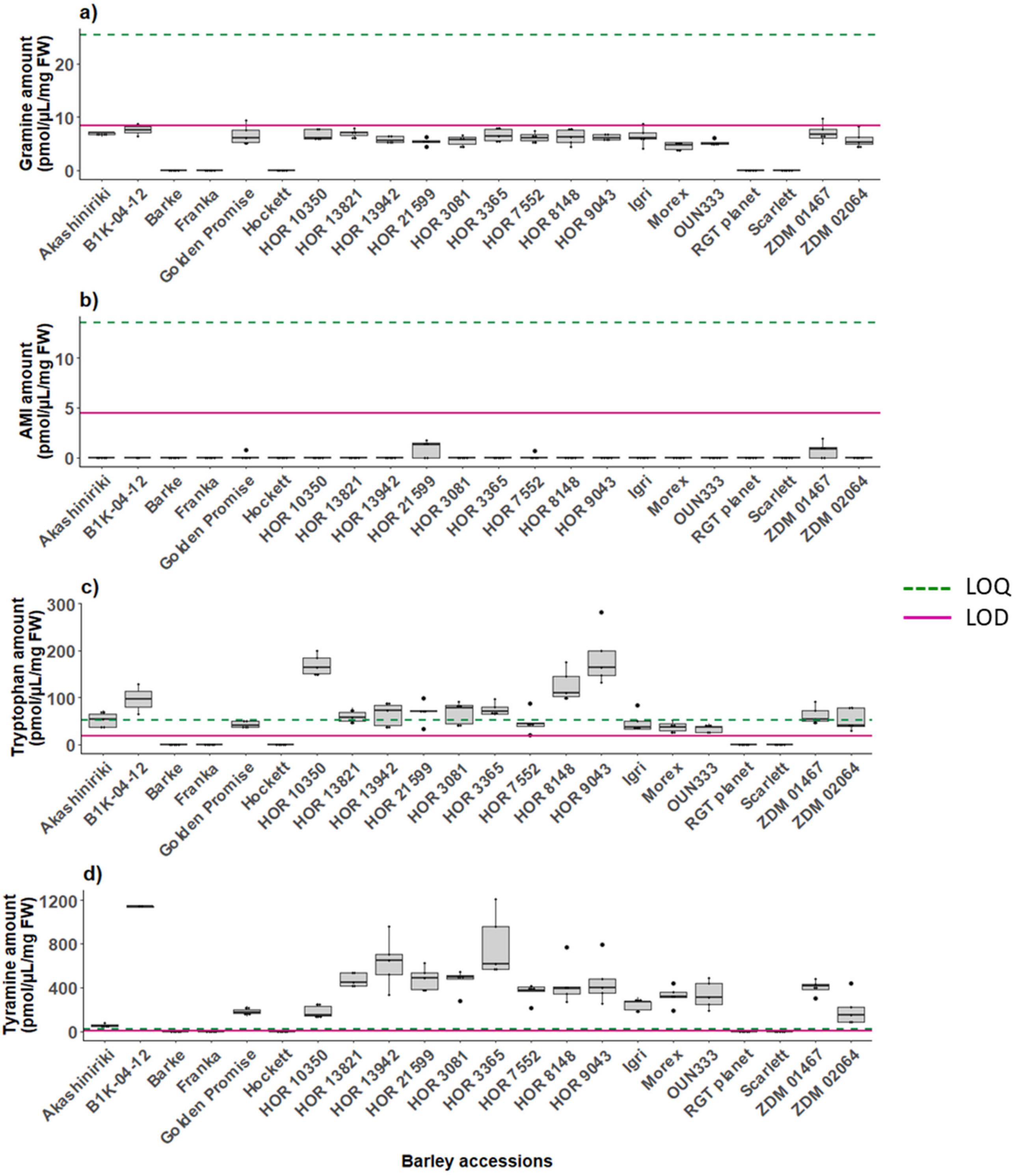

Figure S12: Different analytes production in the roots 8 DAG among 22 accessions of barley (n=6), a) gramine, b) AMI, c) tryptophan, d) tyramine. Graphs report the LOD and LOQ values reported in Table S5.

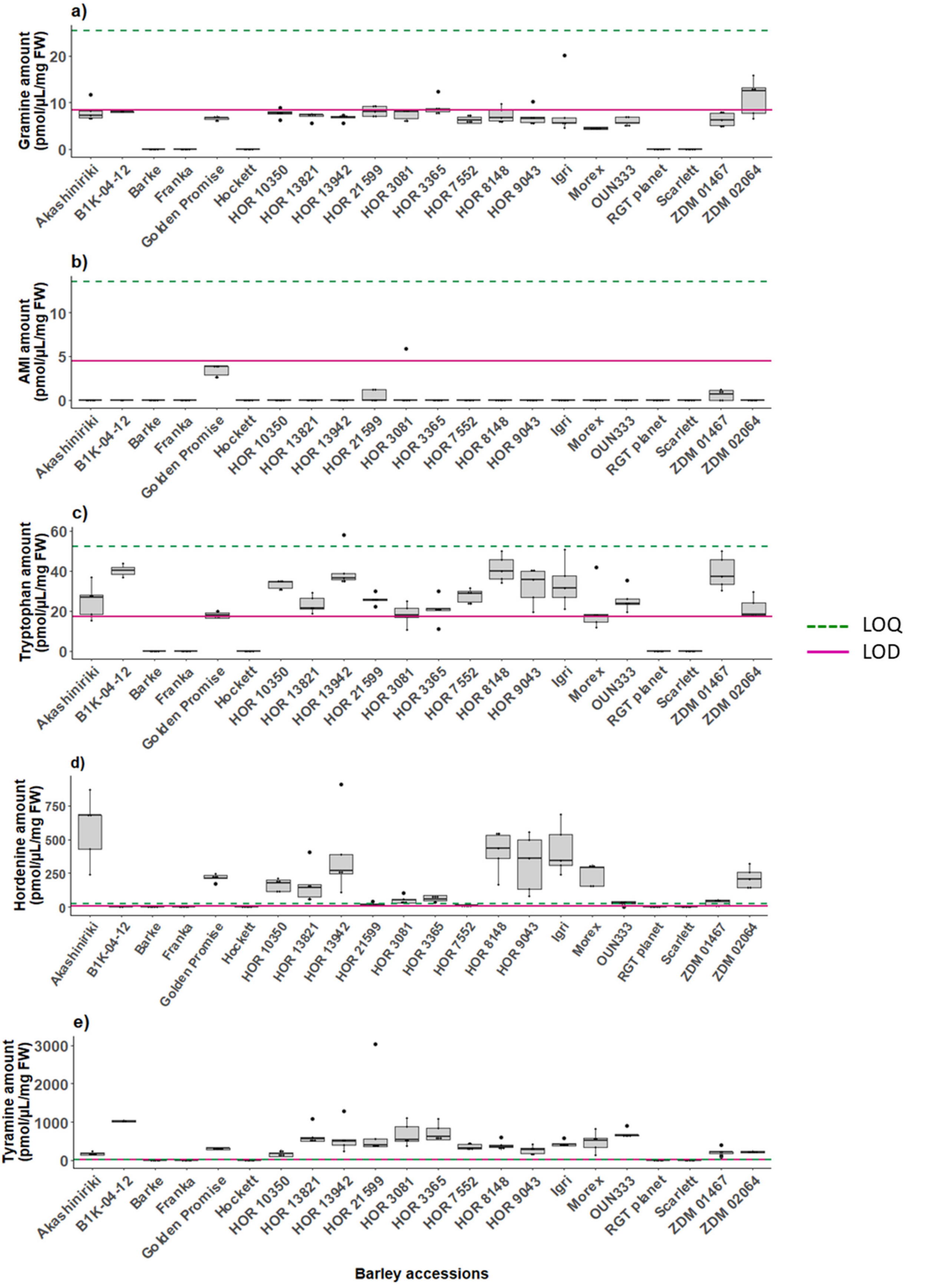

Figure S13: Different analytes production in the roots 11 DAG among 22 accessions of barley (n=6), a) gramine, b) AMI, c) tryptophan, d) hordenine, e) tyramine. Graphs report the LOD and LOQ values reported in Table S5.

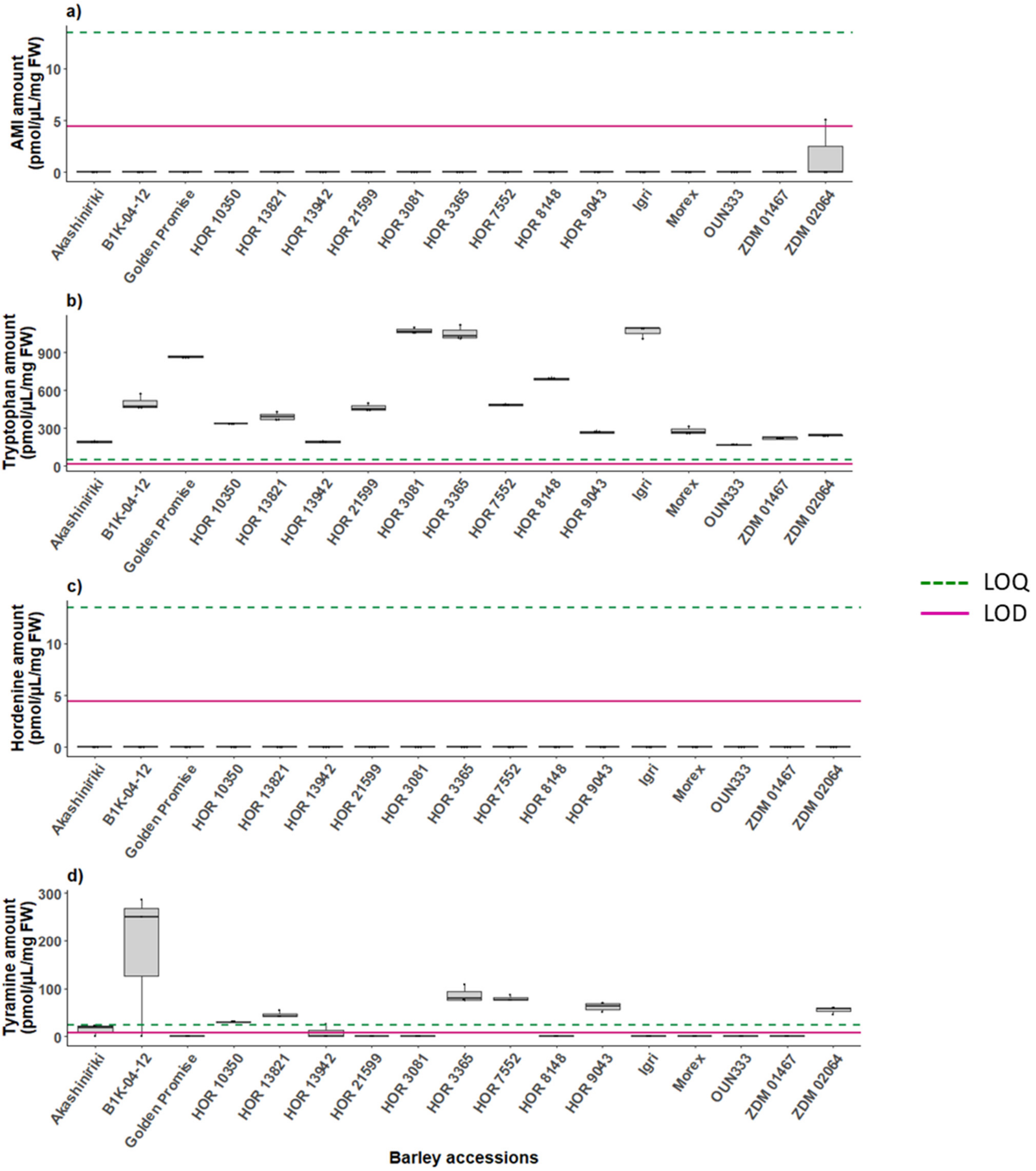

Figure S14: Different analytes production in the kernels of 17 accessions of barley (n=6), a) AMI, b) tryptophan, c) hordenine, d) tyramine. Graphs report the LOD and LOQ values reported in Table S5.

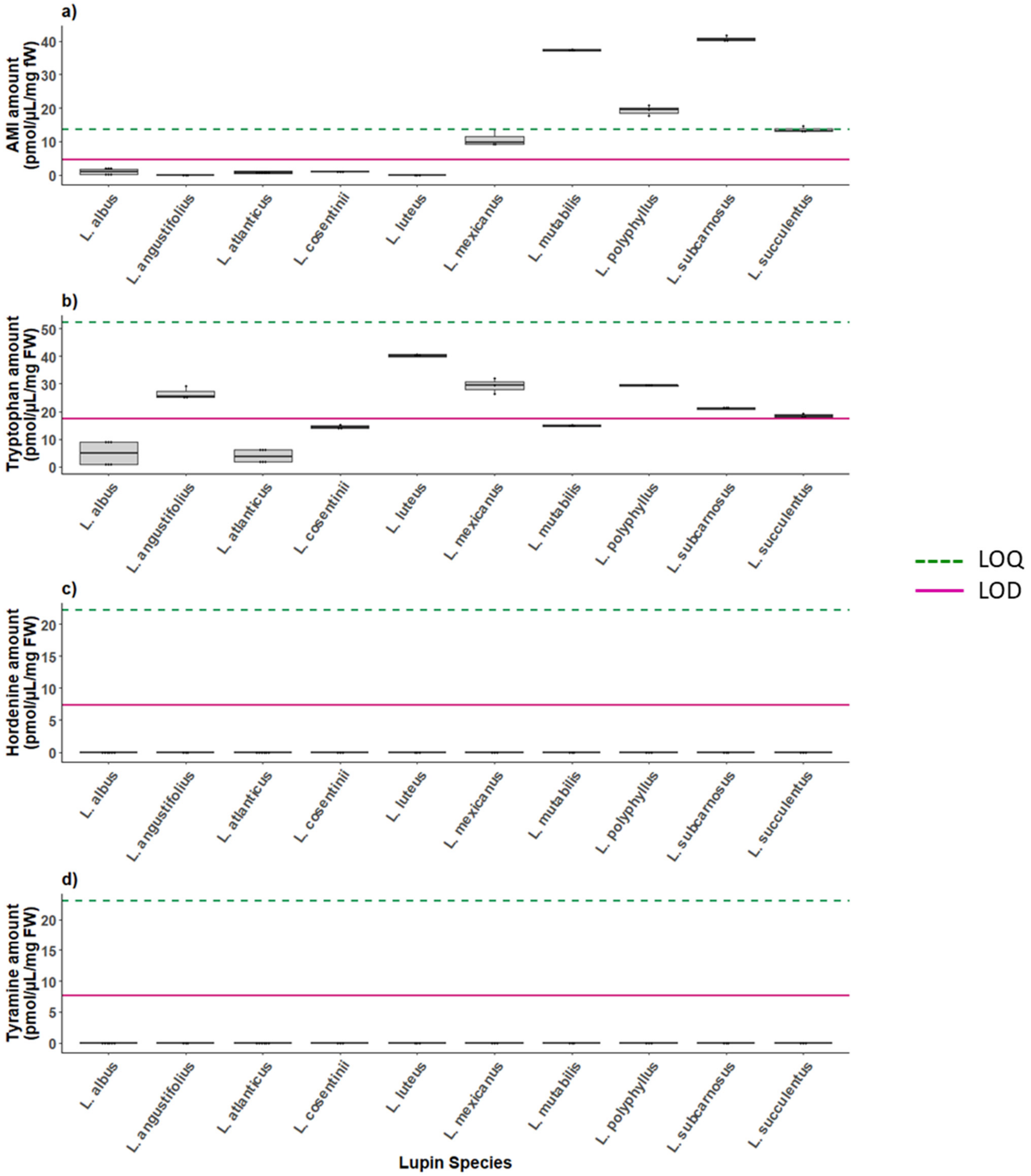

Figure S15: Different analytes production across the seeds of 10 species of lupin (n=6), a) AMI, b) tryptophan, c) hordenine, d) tyramine. Graphs report the LOD and LOQ values reported in Table S5.

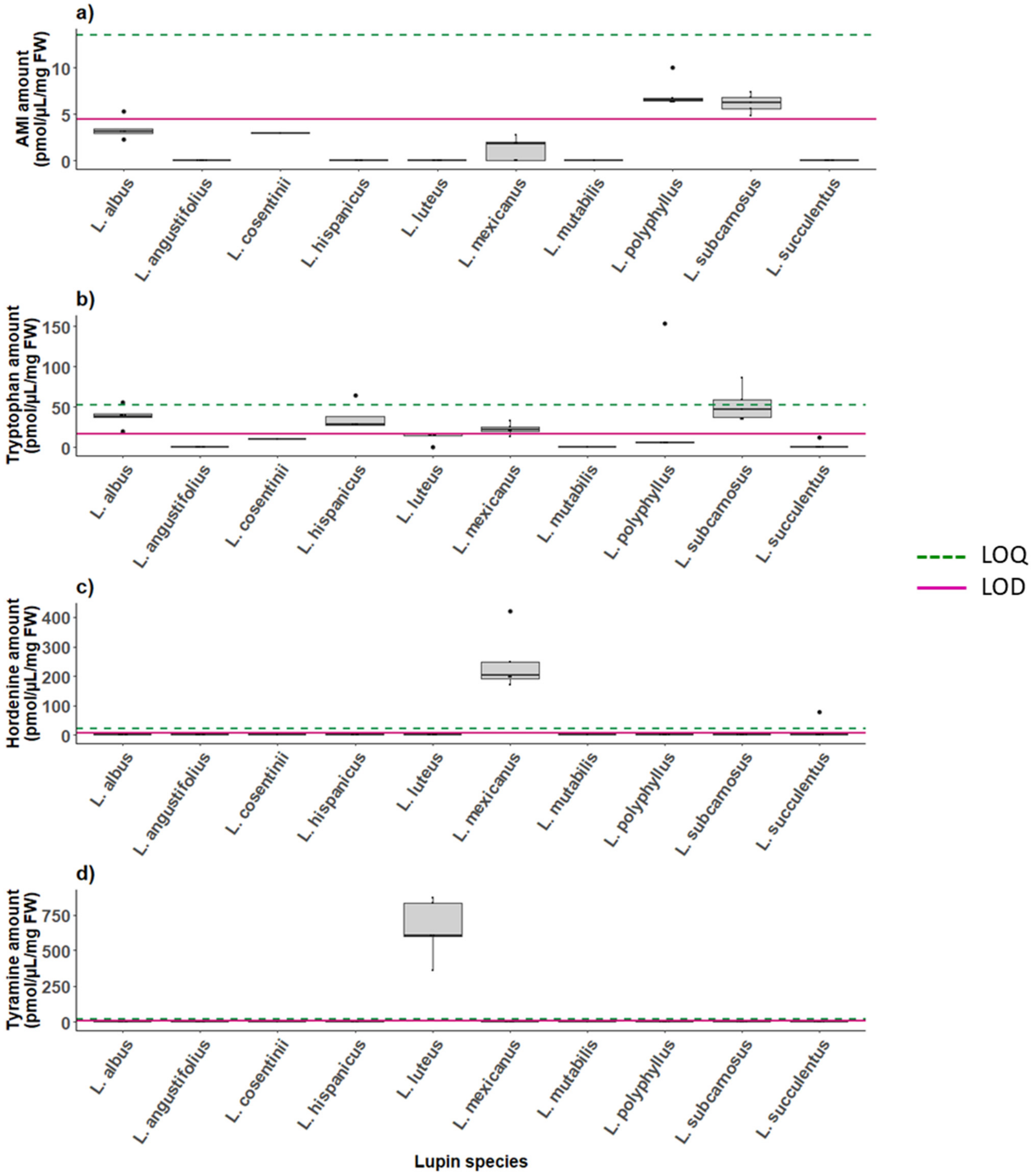

Figure S16: Different analytes production across the leaves of 10 species of lupin (n=6), a) AMI, b) tryptophan, c) hordenine, d) tyramine. Graphs report the LOD and LOQ values reported in Table S5.

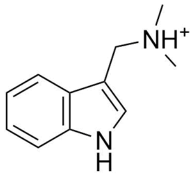

gramine

m/z: 175.12

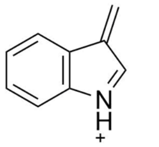

main fragment

m/z: 130.07

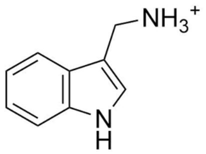

AMI

m/z: 147.09

Figure S17: protonated molecules and main fragment ion for gramine and AMI.
